# The steady progress of targeted therapies, promising advances for lung cancer

**DOI:** 10.3332/ecancer.2016.638

**Published:** 2016-04-28

**Authors:** Lorenzo Bombardelli, Anton Berns

**Affiliations:** 1 Division of Molecular Genetics, The Netherlands Cancer Institute, 1066 CX Amsterdam, The Netherlands; 2 Skolkovo Institute of Science and Technology, Skolkovo Innovation Centre, Building 5, Moscow 143026, Russia

**Keywords:** lung cancer, NSCLC, SCLC, targeted therapies, mouse models, clinical trial, biomarker, drug resistance, genetic screens, EGFR, MAPK pathway, T790M

## Abstract

Lung cancer remains one of the most complex and challenging cancers, being responsible for almost a third of all cancer deaths. This grim picture seems however to be changing, for at least a subset of lung cancers. The number of patients who can benefit from targeted therapies is steadily increasing thanks to the progress made in identifying actionable driver lesions in lung tumours. The success of the latest generation of EGFR and ALK inhibitors in the clinic not only illustrates the value of targeted therapies, but also shows how almost inevitably drug resistance develops. Therefore, more sophisticated approaches are needed to achieve long-term remissions. Although there are still significant barriers to be overcome, technological advances in early detection of relevant mutations and the opportunity to test new drugs in predictive preclinical models justify the hope that we will overcome these obstacles.

## Introduction

The identification of patient-specific, targetable genetic alterations on which tumours depend for their survival is redefining the way patients can be treated [1]. Although chemo- and radiation therapy are still the mainstays, interventions specifically targeting signalling molecules that are mutated or overexpressed in these tumours can have remarkable effects in terms of overall survival (OS), disease-free survival (DFS), and quality of life (QoL). However, it is often difficult to predict whether an individual patient will respond or not. This may be due to the multitude of different subpopulations within a tumour [2], the complexity of signalling pathways that almost invariably include feedback loops, and to the unique genetic makeup of the individual [3].

At the moment, the most prominent benefits of targeted therapies are limited to a small percentage of patients mostly identified by biomarkers that have been correlated with therapy response. Such markers are typically the mutations affecting the gene encoding the drug’s target itself and usually result in a deregulated, always-on growth signal. Although these ‘driver’ mutations are in principle excellent targets/biomarkers, most of the impressive clinical responses to single-agent targeted therapies are limited in time as resistance to the drug almost inevitably ensues. This scenario is a common phenomenon in many different tumour types [4].

Nonetheless, the remarkable regressions achieved by imatinib in CML [5], gefitinib in lung adenocarcinoma [6], and vemurafenib in melanoma [7] illustrate that targeted therapy can be very effective in delaying disease advance and even more so when used early on. These promising observations have further stimulated target gene discovery and drug development facilitated by technological advancements and concomitant cost reduction of high-throughput drug screening and nucleic acids sequencing techniques.

This increased capacity to identify ‘druggable’ cancer mutations allowed for an increasing number of patients to profit from targeted therapies concomitant with a steady progress in understanding the underlying mechanisms of drug resistance. This knowledge is extremely valuable, in that it enables the rational design of combination therapies hitting simultaneously a driver oncogene and a secondary target responsible for the resistance. While this is a promising approach, multiple pathways conveying drug resistance and increased toxicity limit the use of multiple drugs simultaneously [8, 9].

Often tumours escape therapy by restoring the same signalling pathway that is targeted by the drug. It appears that tumours show remarkable addiction to deregulation of a particular pathway and therefore using multiple drugs acting in the same pathway can be more effective without increasing toxicity to normal cells to unacceptable levels. Vemurafenib resistance in melanoma provides a successful example of such an approach: only four years after the first landmark study showing the benefits of vemurafenib in BRAFV600E-mutant melanoma [7], a combination with an MEK inhibitor [10] (MEK activation is a frequent cause of vemurafenib resistance) was approved by the FDA and shown to improve PFS and OS in a 2014 phase III trial [11].

Improved PFS and OS is the ultimate test for a rationally designed, biomarker-based therapy, and the rapid approval of this drug combination illustrates the importance of deciphering the precise mechanisms of drug action and the escape mechanisms which cause it to become ineffective.

The concept of synthetic lethality (a gain or loss of function in one gene makes a tumour cell fully dependent on another gene) offers intriguing possibilities for interventions, as in principle it also allows the treatment of loss-of-function (e.g. tumour suppressor) lesions and limits toxicity to normal cells. An appealing example is the effect of Parp inhibitors in BRCA-deficient tumours [12]. Although this approach is also plagued by escape mechanisms [13], escapes might be less frequent because of the limitations imposed on escapes by the synthetic lethal interaction. The near future will reveal whether the many synthetic lethal interactions that are being revealed by shRNA and Crispr/Cas9 or insertional mutagenesis techniques lead to more effective interventions.

There are other therapeutic options besides targeted therapies that deserve mentioning. Immunotherapy, for example, has finally come to the fore resulting in unprecedented long-lasting remissions in patients with advanced disease [14]. Once again studies in metastatic melanoma have led the field, making it clear that this approach can also be applied successfully to other cancers, especially those with high mutation loads such as lung cancer [15]. Here major developments are already evident and more can be foreseen in the coming decade.

## Current status of translational research in lung cancer

### Basic knowledge

In this review we want to focus on targeted therapies in lung cancer, more specifically non-small cell lung cancer (NSCLC) as targeted therapies and immunotherapy have been most promising with these tumours. We will minimally discuss small cell lung cancer (SCLC), as we recently published an extensive review on SCLC [16]. Furthermore, advances in SCLC have been very limited. The same holds for squamous cell carcinoma of lung (SCC), where the survival benefits of targeted therapies have remained limited to only several months [17].

Thanks to several studies [18, 19, 20, 21] and the Cancer Genome Atlas project [22], we have a detailed insight in the mutational/molecular landscape of the three major histotypes of lung cancer (NSCLC, encompassing adenocarcinoma (ADC), squamous cell carcinoma (SCC), and SCLC). From a molecular point of view, these are very different diseases, each with a unique molecular signature. ADC is dominated by alterations and mutations in *EGFR*, *KRAS*, and other molecules resulting in sustained activation of the MAPK pathway. SCLC is instead characterised by the combined loss of RB, P53, and frequent amplification of *MYC* oncogenes, absence of activation of the MAPK pathway, and a more prominent dependency on the PI3K pathway. SCC often carries mutations in *P53*, *CDKN2A*, genes in the PI3K pathway, and amplification of *SOX2*, *NFE2L2*, *PDGFRA*, and *FGFR1*. Many other recurrent genetic alterations have been identified in each lung cancer type, although their frequency is typically below 5%.

How can we use this knowledge to design effective therapies? We can view the genetic landscape of lung cancer essentially as a comprehensive collection of recurrent genetic events that influence tumour initiation and progression. This gives us the opportunity to identify on which of these events tumour cells depend for their growth and/or survival, try to link different mutations to a common function, or signalling pathway and finally select a target for intervention on that basis. The target chosen might be a mutated driver or a wild-type (wt), non-mutated protein-activated downstream from several of the mutations found in the tumour. In the case of NSCLC we see frequent lesions in the MAPK pathway. These include gain-of-function mutations in EGFR, K-RAS, BRAF, and EML4-ALK, all signifying the importance of the MAPK pathway for this tumour type.

### Resistance to EGFR inhibitors and tumour heterogeneity

A large body of preclinical [23] and clinical evidence has shown that targeting driver oncogenes in NSCLC is indeed a promising strategy towards more effective therapies and the inhibition of EGFR [6, 24] and ALK [25] in NSCLC are the prototypical examples of this approach.

In the case of EGFR, much progress has been made since two landmark studies on NSCLC patients demonstrated that EGFR inhibitors were effective only in tumours harbouring activating mutations in the EGFR gene [6, 24]. Screening NSCLC patients for activating EGFR mutations is now clinical routine and there are three generations of EGFR inhibitors [26, 27] to treat therapy-naive or drug-resistant tumours.

EGFR-mutant lung cancer appears to be a distinct clinical entity and is primarily found in non-smokers [28]. A significant advance in our understanding of the full potential of EGFR inhibitors comes from studying the mechanisms that underlie resistance to such inhibitors. In fact, since the introduction of the first inhibitors, it has become clear that clinical responses obtained in EGFR-mutant sensitive tumours were limited in time and that resistance would inevitably occur [29]. In about 60% of the patients, resistance to EGFR inhibitors in NSCLC is typically associated with the clonal expansion of tumour cells bearing a T790M ‘gatekeeper’ mutation [30]. Interestingly, this mutation is capable of inducing structural changes in the EGFR receptor that prevent its inhibition by first generation inhibitors such as erlotinib and gefitinib, but it also results in slower growth of tumour cells depending on it [31]. A very rare occurring germline T790M mutation predisposes non-smokers to lung cancer, but being a weak oncogene per se it seems to require another EGFR activating mutations to allow tumour development [32].

The question of whether sporadic T790M mutations are already present at the start of the treatment or are acquired following treatment has been around for a long time [33], and the existence of T790M mutation in treatment-naive patients has been demonstrated [34]. While some concerns remain about false-positive rate when employing very sensitive detection techniques, this mutation might exist in a surprising large percentage (up to 80%) of treatment-naive NSCLCs at a very low allelic frequency [35] (below 0.1%). It is noted that even barely detectable mutant cells may be the culprit of resistance has recently also been shown using a high-complexity barcoded library to overcome the limitation of common sequencing technologies. Bhang and colleagues [36] looked at a population of NSCLC cells sensitive to EGFR inhibitors using this technique and found extremely rare (below 0.05%), but pre-existing clones that could expand in the presence of the drug. These clones used the same escape mechanism (MET amplification and epithelial-to-mesenchymal transition) as to some of resistant human tumours. The notion that pre-existing escape mutations can be invariably found when sufficiently sensitive techniques are used aligns with the logic that it is hard to imagine how a mutation causing resistance would be acquired following the treatment with a drug to which the cell is sensitive. Yet, a recent report [37] suggest that T790M mutations might also evolve from cell clones that become resistant to EGFR inhibitors. Summing up on all these observations, it is suggested that genetic heterogeneity within a tumour, whether pre-existing or acquired, is a major obstacle to effective therapies, and that the reliable detection of underrepresented mutations will remain a challenge.

When prior knowledge of a resistance mechanism is available, like in the case of the T790M mutation, having a sufficiently sensitive and robust clinical-grade diagnostic test can help to make a rational decision about a treatment. Digital PCR (ddPCR) is a significant advance in this direction. ddPCR is an increasingly popular and robust methodology that can detect tumour DNA mutations with high sensitivity and specificity compared to other similar techniques. The potential of this technique was first shown in the non-invasive detection of Kras mutations in stool samples of colorectal cancer patients [38] and more recently for the detection of T790M mutations in the circulating tumour DNA of NSCLC patients [39]. Interestingly, this technique allowed detection of the T790M mutation months before the radiological finding of EGFR therapy resistance. Although it will require more optimisation before becoming a clinical routine, accurate knowledge of the T790M mutation status at the time of diagnosis and during the course of therapy will help to guide the choice of first- and second-line therapies. This would in principle allow us to quantify and possibly tackle a major resistance mechanism before it evolves into intractable variants. Alternatively, one might resolve the need for detecting such low-frequency escape mutations by using drugs that targets multiple variants.

Mouse studies of the T790M escape mutant have illustrated another phenomenon that may be of significant importance for how we treat patients over time. The T790M mutant allows for escape from inhibitors that target the L858R mutation in both mouse [40] and human tumours. However, T790M L858R double mutant tumours do show less aggressive growth [31, 41], indicating that escape clones selected as a result of the initial treatment are less fit than the L858R mutant cells. Patients who have become resistant to a targeted therapy are often switched to chemotherapy. T790M-L858R double mutant tumours might respond to chemotherapy for a while, but subsequently react again to the original TKI inhibitor [42] (erlotinib or gefitinib), probably because clones with the original L858R mutation that were not fully extinguished have regained dominance. Two additional observations are relevant noting here. The first is that tumour cells might also become addicted to the conditions imposed by a particular drug and actually respond with tumour cell death upon withdrawal (the benefits of a drug holiday). Similarly, tumours might respond again after the drug holiday when the same drug is given as tumour cells are again pushed out of their newly acquired equilibrium. This emphasises the value of having an array of drugs that target the different variants of an oncoprotein.

Not surprisingly, in the last decade ample resources were invested in developing better and safer inhibitors capable of inhibiting the various EGFR mutants. Afatinib, for example, belongs to the second generation of inhibitors [43] designed to block wt EGFR and several of its mutants such as those with exon19 deletion or with the L858R and T790M substitutions. Afatinib also inhibits the EGFR related receptors HER-2 and HER-4. Activation of HER-2 is a known mechanism of resistance to EGFR inhibitors, so afatinib is in principle a clever drug to tackle two potential resistance mechanisms at once. Large phase III trials showed that afatinib performed well as first-line treatment in tumours bearing exon 19 deleted EGFR [44], but was unexpectedly ineffective on L858R mutant tumours or on gefitinib or erlotinib-pretreated tumours with a predominant T790M mutation. More detailed examination of these large phase III trials showed however that afatinib was active on some of the less frequent activating mutations in EGFR [45].

Fortunately, third-generation inhibitors such as AZD9291 [46] and rociletinib [47] do not seem to have the limitations of afatinib and showed very promising, albeit preliminary, efficacy on T790M mutant tumours, which is the predominant escape mutation. These new drugs have lower affinity for the ubiquitous wt EGFR compared to the tumour-specific mutant EGFR and therefore exhibit a more favourable toxicity profile [26]. Not surprisingly, however, escape mechanisms have been found [48, 49] also against AZD9291, indicating the never-ending need for additional inhibitors or combinations of inhibitors with complementary profiles [49].

The important lesson we have learned from clinical trials and drug resistance experiments is that the combination between the unique biochemical characteristics of each EGFR inhibitor and the genetic heterogeneity of a tumour is the major determinant of response. Given the importance of MAPK pathway to which a substantial fraction of NSCLC is so profoundly addicted, it remains worthwhile to continue developing new inhibitors for EGFR mutants.

### ALK inhibitors: quickly moving forward

The inhibition of ALK is another successful example of molecular therapy in lung cancer. A rearrangement in the genes encoding ALK kinase and EML4 yields an ‘addictive’ oncogenic fusion capable of driving the progression of about 4% of NSCLC [21]. These fusions can be targeted by the specific inhibitor crizotinib and result in tumour regression, longer OS, and improved QoL compared to standard chemotherapy [25]. Other tumour types such as anaplastic large cell lymphoma, inflammatory myofibroblastic tumour, neuroblastoma, are also driven by the same molecular alteration and sensitive to crizotinib [50], emphasising once again how targeting cancer drivers can be a successful approach. These impressive clinical responses were however not durable and multiple ‘gatekeeper’ mutations in the ALK gene caused resistance [51], as was the case with EGFR inhibitors.

There is luckily a second generation of inhibitors which are able to overcome this problem. Ceritinib [52] and alectinib, most notably, have shown efficacy in early clinical trials on crizotinib-resistant lung tumours and show improved capacity to reach brain metastases [53, 52]. Again, preclinical studies have shown that escape mutants can be found also against these second generation drugs [54]. A phase I expansion trial [55] showed that crizotinib is also very active against another target, the less frequent oncogenic fusions (1.7%) involving ROS1 [56], a gene related to ALK. Although the frequency of mutations in ROS1 is low in patients, the significant tumour control obtained with crizotinib indicates that NSCLC patients should be tested for ROS1 rearrangements.

Which ALK inhibitor to administer as first-line or in the resistant diseases is still an open question, and we do not yet have a full patient stratification scheme based on multiple biomarkers, such as ALK mutation type, HGF plasma levels, and MET amplification status. It is clear however, that treating ALK and ROS1 mutant tumours with the appropriate targeted agents substantially improves patient’s outcome. Although it is still a relatively new field (ALK mutations in NSCLC were discovered in 2007 [57] and crizotinib was granted accelerated approval in 2011), ALK inhibitors can be given to lung cancer patients on a biomarker-based approach. Finally, with so many promising second- generation inhibitors in early trials, we can expect that the prognosis for patients with all ALK mutant tumours, not only lung cancer, will keep improving.

### Successful targeting of other rare mutations in NSCLC

The quest to find tumours dominated by a unique molecular alteration that could be targeted as effectively as in the case of EGFR and ALK mutants is a fast evolving field. By sequencing large cohorts of tumours and performing longitudinal studies, we continue to discover small groups of previously overlooked patients who can benefit of targeted therapies. For example, mutations in FGFR1, BRAF, RET, MET, HER-2 occur in about 3% of all NSCLC [21], and since tumours bearing these mutations were already successfully treated with targeted therapies in other types cancers, it has been hypothesised that they might be effective in NSCLC as well. The BRAF inhibitor vemurafenib for example, was tested in a series of non-melanoma tumours [58] including NSCLC. The response rate in BRAF mutant, platinum-pretreated NSCLC patients was 42%, where the expected rate for standard second-line therapy would have been only about 7%.

The preliminary success with vemurafenib is an indication that multiple NSCLC subgroups can benefit from targeted therapies. Whether other targeted drugs will work equally well in NSCLC has still to be demonstrated, because targeted therapies’ efficacy is heavily influenced by the cell of origin [59] and the wiring of the signalling networks, besides the presence of a sensitising driving mutation.

### Emerging new therapies

Among the latest advances in lung cancer, immunotherapy deserves an honorable mention. Immunotherapy has finally arrived to the fore and delivered unprecedented, long lasting remissions in previously intractable diseases such as metastatic melanoma [60] and more recently also NSCLC [61]. These successful therapies are based upon releasing the signals that keep cytotoxic T-cells in check. The effectiveness of such therapies seems to depend on the visibility of the tumour to the immune system, i.e. the presence of neo-antigens and the capacity of the tumour to present them [62]. Lung cancer and melanoma have a high mutation load and therefore a higher probability of having such neoantigens. Recent trials in advanced metastatic lung cancer treated with single checkpoint blocking agents, such as anti-CTLA4 and anti-PD1/PDL1 antibodies [63], have shown impressive efficacy and acceptable toxicity. This is a highly promising field that will likely explode in the years to come.

## Mouse models and technology advances

Molecular diagnostics in lung cancer is continuously advancing thanks to the constant evolution of preclinical models where we can validate targets, investigate underlying mechanisms, and test new drug regimens. Here genetically engineered mouse models (GEMMs) models [64, 65, 66], and patient-derived xenografts (PDXs) [67] of *KRAS*, *EGFR*, and *EML-ALK*-driven tumours have been instrumental in understanding the key aspects of NSCLC biology. In these models one can conduct proof-of-concept experiments, using genetic ablation or specific mutations in a gene to mimic for example its pharmacological inactivation. These approaches are not equivalent as the complete removal of a protein, e.g. a kinase might not always result in the same phenotype as the pharmacological inhibition of the same molecule [68]. Still genetic knockouts are very informative when there is a question of whether a certain signalling molecule has a major impact on tumour initiation and progression.

The *KRAS^G12V^*-driven mouse model of NSCLC [64] recapitulates well the phenotype of human mutant RAS-driven NSCLC that has so far been refractory to most interventions. Targeting directly mutant KRAS with small molecules inhibitors has proven to be very challenging although progress in generating inhibitors for K-ras is being made [69]. Research in this field has therefore largely moved towards blocking effectors or critical signalling nodes downstream of KRAS [70]. This approach was extensively tested in the *KRAS^G12V^*-driven mouse model of NSCLC and the outcome of many experiments all point to a pivotal importance of the MAPK [71, 72] and PI3K pathway [73, 74] in the propagation of mutant KRAS’s oncogenic signals. In particular, RAC-1 [75] and c-Raf [72] appeared to be critical downstream (*of KRAS*) nodes in the MAPK pathway whose inhibition is not toxic *per se*, but blocks proliferation only in *KRAS* mutant tumour cells. Puyol *et al* provided another compelling case of how mouse models can be used to precisely validate a target [76] by showing that Cdk4 but not Cdk2 or Cdk6 inhibition is synthetic lethal with activated mutant KRAS and can eliminate mutant KRAS lung tumours. These results help explain why inhibitors of multiple Cdks failed as generic cancer therapies [77] and suggest that focusing instead on a critical kinase (Cdk4) in a specific context (KRAS mutant NSCLC) might be a better approach.

Here mouse models provided key evidence that we can identify and validate targets in NSCLC whose inhibition is likely to be maximally effective on tumour and minimally toxic on normal tissue. Future trials will need to show whether these findings can be translated to human lung cancer.

GEMM models of lung cancer are in principle also suited to directly test targeted therapies. A systematic review of pharmacological interventions performed on GEMMs of lung cancer [23] suggest that with respect to the most clinically relevant mutations, such as those in EGFR [78] and ALK [79], GEMMs well recapitulated the features of human tumours in terms of response and resistance mechanisms. For example, in the case of the *EGFR^L858R+T790M^* mouse model, resistance because of EGFR T790M could be prevented by a combination of afatinib and cetuximab [65]. GEMMs are also a valuable testing platform in the cases of rare lung cancer mutations, such as those affecting the ALK gene. Here, establishing a sufficient collection of human samples and cell lines might take years, while a mutant ALK GEMM can be exploited as a much faster alternative to gain insight into the underlying signalling mechanisms and test treatment regimens.

Finally, a systematic investigation of the plasma proteome of GEMMs of NSCLC and SCLC [80] showed a great deal of similarity to the human disease, suggesting that they could also be used to search for early detection markers and therapy response monitoring.

Another remarkable advance regards PDX models. Given the access to NSCLC surgical specimens and their propensity to engraft in immunodeficient mice, several groups have been able to establish a sufficiently large collection of PDXs representative of the major molecular subtypes [67] (EGFR, ALK, KRAS mutant). Propagation of human tumours in mice is demanding in terms of effort and costs. Furthermore, not all tumours engraft successfully or can be maintained long-term [81]. It is, however, clear that PDX soften recapitulated well the human disease [82], largely maintained the genetic identity of the donor tumour [67], and more importantly reproduced the response and development of resistance to targeted therapies [82, 81].

Recently Hodgkinson *et al* reported anovel promising PDX technology [83] which provided a solution to a long-standing problem in SCLC, namely the almost complete lack of PDX models because of the scarcity of surgically resected SCLC samples. This type of PDX, called CTX, is based on the efficient capture of viable human circulating tumour cells (CTCs) and their subsequent grafting in nude mice.

Human CTCs engrafted and grew as tumours matching the donor’s histology and response to cisplatin, the standard treatment for SCLC [84]. Technologies to obtain and expand small numbers of viable cancer cells from a blood sample of patients constitute a major advance in translational research, not just the SCLC field. More work is needed to understand the precise relationship between circulating cells and primary tumours, but the evidence so far suggests that CTCs can be used in the early detection of cancer [85], to monitor therapy response, and to predict metastatic spread [86]. Therefore, PDXs and CTXs greatly complement GEMMs: where PDXs/CTXs allows for conducting experiments on relevant human tumour cells in a setting closely resembling a clinical trial, and the GEMMs offer more genetic manipulation opportunities on a larger experiment scale.

## Conclusion

**Figure 1.**
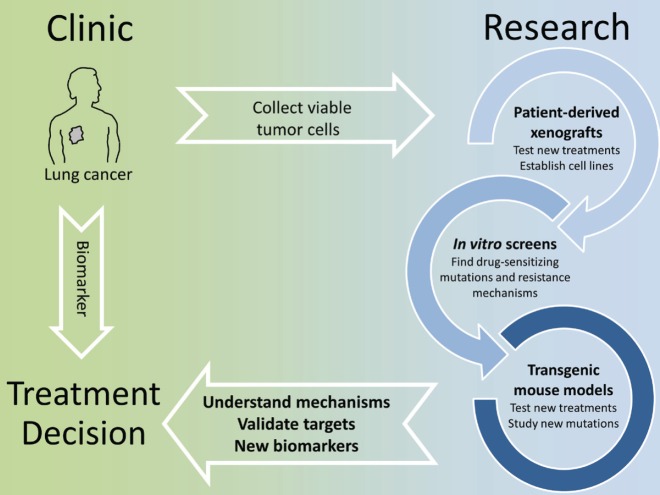
In vitro screens, patient-derived xenografts and transgenic mouse models can be used to model treatment and quickly identify known or new molecular alterations that affect the efficacy of targeted therapies. This knowledge can be translated into biomarkers to select patients eligible for a given therapy and more broadly to understand how tumor genetics evolve under the selective pressure of a drug.

The identification of actionable mutations in lung cancer is significantly changing the way patients are treated. Already the first generation of EGFR inhibitors showed how a biomarker-based therapy, when possible, resulted in an overall better patient outcome. While there is a constant improvement in finding eligible patients by deep sequencing of tumour biopsies and testing a wider panel of markers, the most significant challenge we face at the moment is that therapy-resistant tumours quickly develop with complex genetic profiles thereby making a rational treatment decision very difficult. Even using the latest inhibitors, resistant tumours will remain a moving target and this requires a highly individual approach. Here basic research is invaluable. Thanks to the advances of *in vitro* and *in vivo* screening techniques and the validation of novel intervention protocols in GEMMs and PDXs, we can now quickly gain insight into the mechanisms of drug resistance and how to overcome these. This should translate into clinical trials in which longitudinal monitoring of tumour evolution with concomitant adaptation of intervention strategies would receive increased emphasis (Figure 1).
